# In pursuit of goodness in bioethics: analysis of an exemplary article

**DOI:** 10.1186/s12910-018-0299-9

**Published:** 2018-06-15

**Authors:** Bjørn Hofmann, Morten Magelssen

**Affiliations:** 1 The Institute for the Health Sciences, at the Norwegian University for Science and Technology (NTNU), Gjøvik, Norway; 2 Centre for Medical Ethics, University of Oslo, PO Box 1130, Blindern, N-0318 Oslo, Norway

**Keywords:** Quality, Goodness, Method, Excellence, Exemplary

## Abstract

**Background:**

What is good bioethics? Addressing this question is key for reinforcing and developing the field. In particular, a discussion of potential quality criteria can heighten awareness and contribute to the quality of bioethics publications. Accordingly, the objective of this article is threefold: first, we want to identify a set of criteria for quality in bioethics. Second, we want to illustrate the added value of a novel method: in-depth analysis of a single article with the aim of deriving quality criteria. The third and ultimate goal is to stimulate a broad and vivid debate on goodness in bioethics.

**Methods:**

An initial literature search reveals a range of diverse quality criteria. In order to expand on the realm of such quality criteria, we perform an in-depth analysis of an article that is acclaimed for being exemplary.

**Results:**

The analysis results in eleven specific quality criteria for good bioethics in three categories: argumentative, empirical, and dialectic. Although we do not claim that the identified criteria are universal or absolute, we argue that they are fruitful for fueling a continuous constitutive debate on what is “good bioethics.”

**Conclusion:**

Identifying, debating, refining, and applying such criteria is an important part of defining and improving bioethics.

**Electronic supplementary material:**

The online version of this article (10.1186/s12910-018-0299-9) contains supplementary material, which is available to authorized users.

## Background

What is good bioethics? More specifically, what quality criteria should we apply to this field of ethics? This is the core question in this article. The answers to the question is of great import to all actors in the field, be it editors, researchers, readers, and users of the bioethics literature. They are not only important to decide what will be published and read – but also to what defines the field as such [1].

In order to address the question, we performed an initial literature search^1^ which identified two articles published in the last 10 years whose purpose has been to characterize quality criteria for normative bioethics [1, 2] as well as 24 other articles indirectly identifying quality criteria. The identified criteria have been grouped and displayed in Table 1.

**Table 1:** Quality criteria for normative bioethics proposed in the literature

Argumentative concerns	Dialectic concerns	Pragmatic concerns
• being accurate, consistent, and coherent [2], ensuring integrity, trustworthiness, transparency, and accountability [1], including “rigour and transparency in all literature review processes undertaken in bioethics” [33] • being based on solid philosophy [12] • being principle based [34, 35], being “practical in approach, philosophically well grounded, cross disciplinary”; and being performed by good people [36]	• responding to disagreement by improving understanding [37], • “contributing to the debate on problems people experience in real life and to changing practices” [38], • engaging the public [39], fostering a morally good public deliberation [40], or drawing “attention to the normative underpinnings of global health justice and distribution” [41] • identifying and avoiding “moral fictions” [42], • fostering “sensitivity to the problem of the multiplicity of moral traditions” [43]	• resulting in better health and wellbeing [40], or making the world a better place [44], resulting in changes in practice or policy [1, 45] • empowering action [46], being functional or instrumental [40], facilitating legislation [35], legitimizing governance practices [47], • making clinical medicine better [48], providing “sound action-guiding prescriptions” [49], • advancing “awareness of the sorts of institutional considerations that might lead to a divergence between bioethical analysis and legal [and policy] analysis” [43]; opposing and correcting law [50]; addressing non-ideal circumstances with non-ideal theories in order to contribute to effective policy design [51]. • attending to both the biomedical and existential aspects of illness [52], countering a “a progress and technology-driven model of medicine” [53], appreciating the intrinsic value of human life [54–56].

Many bioethicists may acknowledge and endorse the various criteria of the above list. However, the list has some shortcomings. First, it does not provide an elaborated and agreed-upon list of quality criteria for bioethics. As aptly pointed out by some of the founding fathers of bioethics in the UK, such an assembly of criteria “certainly doesn’t provide a canonical account of what it is to do good medical ethics.” [3] Second, the reason for this may be that goodness can have many meanings, e.g., with respect to *what the criteria are good for*. Some criteria refer to academic rigor, while others refer to adherence to philosophical standards. Third, the criteria may have very different significance depending on the field of bioethics. Some criteria may be more important in philosophical bioethics than in narrative bioethics, sociological bioethics or feminist bioethics and vice versa. Fourth, quality criteria may also be directed towards a wide range of (external) goals, and be chosen from whether they promote such goals. These may amongst many others be: raising awareness, informing policy, challenging or reinforcing theoretical perspectives or practical norms, provoking change, attracting attention, educating professionals, policy makers or the public.

Hence, markers of goodness in bioethics may differ depending on form, content, sector, purpose, and context. Nonetheless, the criteria may coincide. An academically well written paper may adhere to traditional philosophical standards of coherence and consistency, be theoretically well supported, empirically well informed, relevant to a wide spectrum of disciplines in bioethics, be thought provoking to the general public, useful to policy makers etc.

To address all these aspects of goodness is beyond the scope of one article. Here we will mainly focus on applied philosophical bioethics, and in particular how well bioethics is able to illuminate the question at hand and inform and promote open and transparent decision-making. Despite these limitations, we hope that the results may have some relevance to other purposes, contexts, and disciplines of bioethics as well. Moreover, we hope that the overview provides a basis for reflection and improvement.

While quality norms have been proposed for ethics in specific fields, such as in ethical analysis in health technology assessment [4], for formal assessment of the clinical medical ethics literature [5, 6], for systematic reviews of arguments and reasons [7], for synthesizing ethics literature [8], for assessing expertise for decision making [9], for assessing the quality of case consultations [10], as well as for evaluating ethics in health care organizations [11], there are so far no general, clear, and agreed norms for what constitutes “good bioethics.” This may of course be disturbing, as every accepted and respectable field of expertise is expected to have a fairly clear set of constitutive norms. However, it may also express the characteristics of a dynamic and evolving field.

The next step in order to provide a coherent, consistent, and applicable set of quality criteria for bioethics could be to debate, eliminate, revise, and refine the already identified quality criteria for bioethics. However, we believe that more work is needed before we can start such an endeavor. There may be many more quality criteria to identify before we can begin the analysis and synthesis of general quality criteria for bioethics.^2^ Besides, while the latter work is the task of a larger group of scholars, the former can be done by single researchers. Moreover, some would argue that synthesizing general quality criteria for a diverse field such as bioethics is impossible. Be that as it may, it can still be of great methodological, practical, and positioning value to elaborate on quality criteria for bioethics. Accordingly, the objective of this article is to formulate candidate criteria for quality in bioethics.

In this article we therefore set out to identify other relevant quality criteria for good bioethics than those identified by our initial literature search.

## Methods

To do so, we apply a rather innovative method, as we perform an in-depth analysis of one specific article. The article was selected on basis of being acclaimed for being exemplary by a scholar who has been especially preoccupied with quality in bioethics, both in publications [12], and as the Editor-in-Chief of a renowned bioethics journal (Journal of Medical Ethics), and who praised the article for being “in the tradition of good medical ethics” which “challenges conventional societal stereotypes, calling for reasons to justify current practice and attitudes. It also provides excellent, coherent arguments that are a fine example of method in bioethics” [13].

The acclaimed article which we investigate is Ole Martin Moen’s *Is Prostitution Harmful?*^3^ [14] The article exemplifies formal qualities (coherent arguments) as well as merits of implication (challenging societal stereotypes, practices, or attitudes). It was given its due place of prominence as a Feature article and three invited commentaries [15–17], in addition to two responses by the author [18, 19], were printed alongside it. We want to use this exemplary case to explore and discuss other characteristics of “good bioethics.”^4^ We also address some general issues with a pervasive position in modern bioethics of which this article is an example and which has produced a wide range of scholarly publications, attained broad public interest, and tends to strongly influence health policy, i.e., radical consequentialist bioethics [20].

We agree with the Editor-in-Chief that the article is written in a clear language and that it challenges conventional stereotypes. A close reading of the article also brings to light a series of other quality criteria for “good bioethics,” such as:

- Presenting clear objectives
- Refraining from drawing conclusions beyond premises or objectives
- Not taking crucial or controversial premises for granted
- Assessing the truth of the premises accurately, in particular by rigorous quality assessment of empirical evidence
- Using warranted or well supported examples, analogies, and thought experiments
- Distinguishing empirical and normative arguments in a clear way
- Making underlying theoretical assumptions explicit
- Avoiding theoretical blind spots

Notably, our aim is not to enter the debate on the topic of the article selected for scrutiny, i.e., prostitution, but rather to continue and contribute to the debate on what is good bioethics.

## Results

The selected article sets out to challenge the conventional conception that prostitution is harmful. It does so by challenging nine common arguments why prostitution is harmful. After undermining these arguments, the author concludes that “prostitution is no more harmful than a long line of occupations that we commonly accept without hesitation.” This exemplifies a characteristic of good bioethics: *To present counterarguments (in a manner that opponents can accept) and to counter these arguments with clear (counter-counter) arguments*.

However, the objective of the selected article goes beyond showing that prostitution is not harmful. The author wants to argue that in the same manner that we have changed our attitudes to and legislation on slavery and homosexuality, “what we need is a shift in our social and legal treatment of prostitutes.” [14] Hence, there is both an ethical and a legislative argument, which are intertwined. This exemplifies a feature acclaimed by the Editor-in-Chief: The article is intended to challenge existing conceptions and call for changes.

As we interpret it, the overall ethical argument of the article is as follows: casual sex is morally acceptable, and therefore prostitution is acceptable. A premise for this is that «sexual encounters need not be deeply personal and emotional in order to be acceptable» and that casual sex is in some sense equivalent to prostitution.

The regulative aim comes out in another version of the argument (formed as a conditional): “[I]f casual sex is acceptable, then we have few or no reasons to reject prostitution.” This is argued for by way of the following conditional: “if we accept the increasingly common view that casual sex is not harmful, we should accept that neither is prostitution.”

The main argument of the article appears to have the following structure:

P1: Sex without romantic significance is possible.

P2: Sex without romantic significance is not a problem (i.e., is not harmful).

P3: Prostitution is equivalent with sex without romantic significance.

C1: Prostitution is not a problem.

The regulative version of the argument has the following structure:

P4: Prostitution legislation presupposes that prostitution is harmful.

P5: Prostitution is not harmful.

C2: Prostitution legislation is not warranted.

### Are the premises true?

One criterion for a valid (and good) philosophical argument is that the premises are true. Let us therefore investigate the premises of the main argument.

### Sex without romantic significance (P1 & P2)

The first premise (P1) is that sex without romantic significance is possible. However, the article gives no compelling evidence that P1 is true. It assures that “more and more of us … believe that sexual encounters need not be deeply personal and emotional” but no empirical evidence is given beyond belief. Moen states that “I will take for granted that all sex between a prostitute and a client is sex without romantic significance”, but it is unclear what romantic significance means. The author gives a clue when he explains that casual sex is like “occasionally catching a cheap hotdog on the run,” and just as such “casual eating” does not destroy the culinary significance of other meals, casual sex will not destroy the capacity for romantically significant sex.^5^ However, if sex without romantic significance was only genital pleasure like “a cheap hotdog on the run,” why do we need another person? If prostitution is non-relational beyond the exchange of genital pleasure and money, why do we need a human partner in the first place? Can’t we just use a machine? The point here is not to enter the debate on casual sex or prostitution, but only to point out the importance of defining one’s key terms, in this case “romantic significance,” and to point out that by excluding definitions of key terms (“without romantic significance”) we may do no “heavy philosophical lifting” at all.

Moen argues for the second premise (P2) in two ways. First, by referring to what he calls the “weak significance view,” according to which “sex need not always be romantically significant in order to be permissible.” Hence, sex can be permissible if it is without romantic significance. However, instead of showing this, the author takes this for granted (see above quote).

Second, the author claims that the view that sex without romantic significance is not harmful is “increasingly common.” This is not support for the truth value of this premise either. However, the author takes this important premise for granted: “I take for granted here that casual sex is not harmful” and “sex without romantic significance is not per se a problem.” This certainly counters those who think that it is impossible to have sex without some personal elements, and that this can cause problems of moral significance. However, no supportive evidence is given for these premises. It is not in itself problematic to presuppose certain premises, but it is important then to keep in mind – and to remind the reader throughout – that the truth and relevance of any conclusions drawn are conditional on the veracity of the controversial premise.

### The casual sex – prostitution equivalence (P3)

What then about the third premise – is this true? Or more precisely, is prostitution equivalent to casual sex? Formally, Moen phrases this as a conditional: “If casual sex is not harmful, however, I argue that prostitution … is not harmful either.” As the author takes for granted that casual sex is not harmful, prostitution is not either, given the conditional. However, this presupposes what is to be shown, i.e., that prostitution is not harmful, and thereby is a *petitio principii*, or it is a *reductio ad absurdum* for those who reject the first part of the conditional (as the author admits). In either case the argument comes to a full stop. Hence, to make the argument do any work at all, this premise must establish that prostitution is analogous to casual sex, which is also how the critics have interpreted the argument [15–17].

One way to find out whether prostitution is like casual sex would be to ask people that engage in both practices. While there may be few studies asking explicitly about the analogy of casual sex and prostitution, several studies investigate the motives and emotions of persons involved in prostitution [21–23]. Reports on emotional labor, stress, and exhaustion [23, 24] appear relevant to support or assess the analogy, but no such evidence is provided in the article. Moreover, the monetary transaction characteristic of prostitution may be a morally relevant difference between casual sex and prostitution; e.g., an influential line of argument claims that certain goods, such as sex, can be corrupted by being treated as commodities [25].

The point here is not to enter the discussion on the experiences of casual sex and prostitution. It is rather that premises must be backed by evidence. In this article, it is not clear that the equivalence or analogy (which is crucial to the argument) is good.

This illustrates that while there is a fairly clear structure of the argument and the argument is framed in a clear manner (formal feature of good bioethics) the truth values of the premises are partly taken for granted and partly insufficiently argued for. While the premises might appear plausible and attractive if you follow a specific ethical theory, they might not if you don’t. Thereby, a crucial part of the ethical debate (P2) is bypassed (or an obstacle for progress in the field is overcome, depending on your position). This illustrates a general point: *What you consider to be a good argument, can very much depend on your explicit or implicit theoretical commitments*. Let us now examine the regulative framing of the argument.

### Prostitution is not harmful (P5)

Moen argues for the second premise in the regulative version of the argument (P5) by empirically undermining no less than nine arguments for prostitution being harmful. Table 2 gives an overview over the counterarguments and their main content.

**Table 2:** Overview of the arguments and counterarguments that prostitution is harmful

Argument (that prostitution is harmful)	Counterargument
The correlation with psychological problems argument P1: That which leads to psychological problems is harmful. P2: Prostitution leads to psychological problems. C: Prostitution is harmful.	Correlation is not causation: Correlation alone is not sufficient to conclude that prostitution leads to psychological problems. (Analogy of homosexuality)
The correlation with danger argument P1: That which is dangerous is harmful. P2: Prostitution is dangerous. C: Prostitution is harmful.	Correlation is not causation: Correlation alone is not sufficient to conclude that prostitution leads to harm. (Analogy of homosexuality)
The objectification argument P1: That which involves objectification is harmful. P2: Prostitution involves objectification. C: Prostitution is harmful.	It is not clear that prostitution involves objectification. Even if it does, the objectification might not be of a harmful sort. (Analogy of marriage and newspaper delivery man)
The exploitation argument P1: That which involves exploitation is harmful. P2: Prostitution involves exploitation. C: Prostitution is harmful.	Some studies show that prostitutes earn more than other women in the same group. Some studies show that pimps do not earn very much. Exploitation exists in other businesses as well. (Analogy of luxury prostitute)
The male dominance argument P1: That which involves male dominance is harmful. P2: Prostitution involves male dominance. C: Prostitution is harmful.	Prostitution does not necessarily involve male dominance. Calling prostitution ‘degrading’ begs the question rather than showing it to be harmful.
The economic dominance argument P1: That which involves economic dominance is harmful. P2: Prostitution involves economic dominance. C: Prostitution is harmful	Economic dominance (in various forms) is common, and not specific to prostitution. (Analogies of grocery store owner, drug dealer, air traveler, hamburger buyer)
The selling one’s body argument P1: That which involves selling one’s body is harmful. P2: Prostitution involves selling one’s body. C: Prostitution is harmful.	Many other professionals also sell their body without thereby necessarily being harmed. (Analogy of counsellors, dancers, masseuses, sumo wrestlers, football players, colonoscopy ‘artists’)
The habitual faking argument P1: That which involves habitual faking is harmful. P2: Prostitution involves habitual faking. C: Prostitution is harmful.	There are limits to how much faking is required. (Analogy of actress)
The selling one’s soul argument P1: That which involves selling one’s soul is harmful. P2: Prostitution involves selling one’s soul. C: Prostitution is harmful.	The argument does not hold on basis of accepting the weak significance view of sex (without evidence). (Analogy of friendship and philosophy professor)

Again, the point here is not to enter the debates on prostitution, but only to tease out some general aspects of ethical methodology and its goodness. One of the characteristics of several of the counterarguments is that they point out that correlation is not causation and demand proof that a causal connection exists. While it is of course true that correlation does not imply causation, an excessive insistence on conclusive proof would be an unreasonable demand; for instance, if the same requirement was applied to health care, it would have wide-reaching implications: Taken seriously, we ought then to stop a wide range of health services, such as breast cancer and prostate cancer screening, which are health services based on associations.

On the other hand, that prostitution can sometimes appear to be beneficial (as argued by the author) might also only be due to a correlation and not causation. Moen appears to demand a causal connection between prostitution and harm, but only an association between prostitution and net benefit. This points to two quality criteria concerning ‘double standards’: First, *one should apply the same standard of argument or principle consistently throughout one’s own reasoning*. Second, *one should not demand of opponents a higher standard of empirical justification than that to which one is prepared to adhere to oneself*.

Despite all counter-arguments undermining the claim that prostitution is harmful, the author accepts that prostitution can be harmful. However, the harm to prostitutes does not consist in “something intrinsic to prostitution, but in contingent external factors.” This leads Moen to engage in a risk-benefit analysis, where he comes out in favor of prostitution. However, rather than referring to overall benefits and risks, as well as corresponding probabilities, which would be expected in a consequentialist analysis, Moen uses the example case of Caroline, who enjoys sex and wants to buy a dish-washer, as well as other professions where risks are accepted, such as professional boxers, to make his point. Moreover, the author accepts that prostitution implies high(er) risks of catching sexually transmitted diseases, might lead to negative psychological effect, and is incompatible with sexually monogamous relationships. However, Moen considers these to be factors that “appear to be present regardless of our social or legal treatment of prostitution, and as such, they are genuine downsides to selling sex.” He admits that these may count as harms and that they may “aggregate to become significant,” but “the sum would be reasonably low.” It is not clear from the empirical argument how this overall assessment is made, which directs our attention to another quality criterion of bioethics, i.e., *stringency in applying methodology*. When doing a cost-benefit-analysis, one “should then compare the sum of total costs and benefits in prostitution with the sum of total costs and benefits in alternative occupations” in a transparent and sufficiently comprehensive way.

This also relates to yet another quality criterion in bioethics, i.e., *normative extrapolation*. The article goes beyond just stating that the prostitution is harmful argument does not hold – it also draws normative conclusions from this with respect to regulation of prostitution. Indeed, the author argues for “a shift in our social and legal treatment of prostitutes.” However, he does so by claiming that “it appears that for some—say, those who accept casual sex, have a high sex drive, need money and are able to work in a safe environment—selling sex could be a prudent option. If this is correct, we must concede that it might be rational to engage in prostitution, and for some, irrational to opt out of it.” However, being able to identify special cases where prostitution may not be harmful, does not provide a knock-down argument for permitting prostitution as little as being able to identify one person who in sum may benefit from slavery is a convincing argument for permitting slavery. Hence, a criterion for “good bioethics” appears to be *not to draw normative conclusions beyond the limitations or premises of the argument*.

### “Empirical through and through”

Let us now turn to another formal aspect of bioethics. What type of argument is the author presenting in the article? He uses analogies, conditionals, conceptual analysis, and refers to the logical structure of arguments. However, in a reply to McDougall’s comments on this matter [16], the author claims that his argument is empirical: “My argument is empirical through and through” [18]. This raises two questions: 1) What is the quality of the empirical argument? 2) Is the argument truly empirical?

The empirical arguments appear to be selective. For instance, with respect to the points that casual sex and prostitution can be without romantic or relational significance, there are relevant articles that effectively undermine this [26] not mentioned in the article. Moreover, when countering research in the field arguing that “Sex work is an extremely dangerous profession,” [27] one would expect solid evidence in empirical studies. Moen dismisses studies showing correlations with arguing that they are not causation. However, studies with the optimal designs to demonstrate causal connections, e.g., randomized controlled trials, may not be possible to perform in this field for ethical and practical reasons. This points to two important quality criteria in empirical ethical argumentation: *Quality assessment of the empirical studies included in the argument* as well as *systematic review of the crucial empirical issues following quality criteria* (see below).

When arguing against the exploitation argument (see Table 2), the author omits to discuss any of the research reporting threats, violence and abuse of prostitutes by pimps (see, e.g., [28]). Instead, he is content to discuss that prostitutes earn much more than their group and that pimps do not “exploit prostitutes to the extent that we often assume.” To corroborate the latter claim Moen refers to two selected empirical studies; he argues as follows: “Shyamala Nagaraj and Siti Rohani Yahaya, studying non-Western prostitution, found that in Malaysia, prostitutes on average share 2% of their income with pimps (see figures in Edlund and Korn).” However, what Edlund and Korn write is: “Nagaraj and Yahya (1995), studying 44 prostitutes in Malaysia in the early 1990s, found that pimp fees amounted to less than 2 percent of what the prostitutes earned net of tips.” [29] That the pimps of 44 prostitutes in Malaysia on average did not make huge profits during the early 1990s is hardly solid evidence against the exploitation argument.

The author also refers to a study by Lillard who “found that less than 6% of Los Angeles’ prostitutes share income with a pimp.” This corresponds well with what is written in Edlund and Korn: “For instance, Lillard et al. (1995) found that less than 6 percent of Los Angeles street prostitutes surveyed in 1990 and 1991 shared income with a pimp.” [29] However, the original study is not accessible and it is unclear how many informants were included and the selection criteria. Even if the referred studies from Malaysia and LA from the beginning of the 1990s were both of high quality and valid for the situation in these places more than 20 years later, it is far from obvious that they are of any relevance to other places. Pimp-prostitute relations in Malaysia and LA more than 20 years ago may have little bearing on present prostitution in London, Oslo, or Mombasa.

With regard to the second question, it is not clear that the argument is “empirical through and through.” The author wants to do much more than to debunk the claim that prostitution is harmful, a claim that he at the end accepts has some merit, but not in an overall cost-benefit analysis. He wants to alter our social and legal treatment of prostitutes and prostitution. No doubt, empirical arguments are important in normative debates. By showing that one empirical premise of a normative argument is false, one can undermine the normative argument. However, this presupposes that the empirical argument a) is truly empirical, b) is of good quality, and c) is relevant for the premise. Replacing or blending in analogies and thought experiments in empirical arguments may reduce the quality of such arguments. Hence, another quality criterion for good bioethics may be to *keep arguments straight*, e.g., to keep empirical arguments empirical, and conceptual analysis conceptual.

The point is that if ethical arguments are claimed to be empirical, they should be truly empirical and the quality of the empirical evidence is crucial for the goodness of bioethics. Anecdotal evidence, selection (cherry-picking), and ignorance of standard quality criteria for empirical studies reduce the quality of the ethical arguments.

### Clear and transparent assumptions

The exemplary article makes a series of assumptions and has a number of delimitations, as will any article in normative bioethics. One apparent assumption is that the only morally relevant question for the judgement of whether prostitution (or casual sex) is acceptable is whether it is harmful. Moen equates “not harmful” with “no problem” and “acceptable.” As stated explicitly: “I deal solely with issues concerning well-being and harm.” By this a series of other potentially relevant objections to legalizing prostitution are excluded. Moreover, one may ask what is meant with “well-being” and dispute the definition of “harm.” Another example is the premise that arguments for legislation presupposes prostitution to be harmful (P4). Although harm frequently has been used as one element of the argument for legislative measures towards prostitution, this does not make the premise true as there may be many other reasons why one may want to regulate prostitution by law. Hence, while the acclaimed article may enlighten a subtopic of the debate (i.e., the relationship between prostitution and harm), it may also result in superficial analysis, especially when the assumptions are not explicitly taken into account in the conclusion (or its implications). In the case of social and legal treatment of prostitution, there may be a range of other relevant issues, which are ignored, and when commented on dismissed [18].

Another related assumption of the paper is that it presupposes a specific type of ethical realism: “I presuppose, in other words, a certain objectivism about harm.” This assumption is not brought to attention or included in the conclusion either; it remains unclear what precisely the assumption entails and what kind of counter-arguments it is intended to exclude from consideration.

A third assumption is the content of one of the premises of the argument (P3, above): “I will take for granted that all sex between a prostitute and a client is sex without romantic significance.” As pointed out, this to a large extent assumes what should be shown (*petitio principii)*.

The article’s relation to consequentialism is also muddled. While the author’s acknowledgment that his “argument is likely to do more work for consequentialists than for deontologists” hints that the argument builds on this specific theoretical basis, the allegiance is never declared explicitly. Nor are the normative implications of such an allegiance discussed; are there for instance counter-arguments that would have had greater traction within deontological or virtue ethical frameworks (as seems likely)?

Hence, in a harm-objectivist, consequentialist perspective with a specific view on sex and prostitution, the argument makes good sense. However, as pointed out, the article clearly appears to aspire to normative force also beyond such specific and controversial assumptions.

This leads us to another relevant quality criterion: *Good bioethics makes clear all its important and/or contestable assumptions, and takes these into account explicitly when discussing and drawing its conclusion, so that the limitations of the conclusion come out clear*. To this one could also add: *Good bioethics does not pose or indicate conclusions that do not follow from its analysis*, i.e., it avoids unwarranted extrapolation or generalization. This points back to Aristotle’s *enthymeme*, that is, as an argument with an unstated premise.

The latter point is spurred by the following: Given the premise that casual sex is acceptable, the author argues that “we have no reason to reject prostitution.” However, what he ends showing is that “it appears that for some—say, those who accept casual sex, have a high sex drive, need money and are able to work in a safe environment—selling sex could be a prudent option.” Hence, there are some persons for whom prostitution is not harmful – therefore prostitution is acceptable. No doubt some persons find pleasure in being beaten or cut, but this does not make beating or cutting people acceptable. There seems to be a (hidden) assumption here that if you can find some persons for whom some activity is not harmful, then the activity is acceptable.

### Benefiting from counterarguments

Several writers commented on Moen’s article [15–17], and Moen also replied to the critiques [18, 19]. This is not to enter the details of the comments and replies. However, some general lessons can be learned from the interchange.

Some commentators [15, 17] target the premise that sex without romantic significance is not a problem (P2). However, as the author appears to take this premise for granted (“sex without romantic significance is not per se a problem”) he is not willing to address the critique. In this, the article seems to fail to acknowledge its own statement “If casual sex is problematic, therefore, so is prostitution.” Moreover, this appears to be a missed opportunity to explicate and strengthen a contested yet crucial premise. Another goodness criterion, then, would appear to be the *ability and willingness to examine, expand on and justify controversial premises in the argument when challenged*.

Relatedly, as a Feature article Moen’s article was criticized from various fields, but Moen finds that the objections have no sway. A goodness criterion for bioethics following from this can be to *take into account objections and counterarguments even if they come from scholarly fields and traditions different from one’s own*, as this may facilitate dialogue and spur progress in the field. As a case in point, consider Westin’s insistence that the personality of the agents engaging in buying and selling sex be taken into account. She asks, “How can we know what harm is when we do not know what it is to be this particular agent?” In the same vein, she stresses that an adequate moral analysis, “requires understanding the ontology of the human agent”. Moen answers that he is “puzzled as to what specifically Westin is looking for.” Although there is a regrettable vagueness to Westin’s misgivings, this confrontation can be construed as a clash between different philosophical traditions, and one wonders whether fruitful dialogue could have been possible and might have enriched our understanding of the issue at stake.

## Discussion

### Proposed quality criteria

We set out acknowledging the merits of this acclaimed article in bioethics that were pointed out by the Editor-in-Chief. By a closer analysis of the content, we have identified a series of additional quality criteria that could be crucial for “good bioethics.” The 11 quality criteria we have identified are grouped and presented in Table 3:

**Table 3:** Eleven quality criteria for bioethics derived from an in-depth analysis of Moen’s *Is Prostitution Harmful?*

Argumentative concerns	1. Presenting counter-arguments in a manner that opponents can accept and countering these arguments with clear (counter-counter) arguments
	2. Underpinning adequately the premises employed, especially those that are controversial or essential to the argument
	3. Avoiding double standards by applying the same standard of argument or principle consistently throughout one’s own reasoning, and not demanding a higher standard of opponents
	4. Introducing only relevant examples, analogies and thought experiments and not substituting these where other kinds of argumentation (empirical or normative) are required
	5. Fostering transparency and explicitness about crucial theoretical assumptions and definitions, including showing explicitly how the conclusions drawn rely on these assumptions
	6. Refraining from drawing normative conclusions beyond the limitations or premises of the argument, i.e., avoiding unwarranted extrapolation or generalization
Empirical concerns	7. Ensuring that the evidence for empirical premises is of good quality according to standard criteria for empirical evidence of the relevant kinds
	8. Keeping the distinction between empirical and normative arguments clear
Dialectic concerns	9. Responding to challenges by examining, expanding on and justifying controversial premises in the argument
	10. Taking into account also objections and counterarguments from outside one’s scholarly field and tradition
	11. Openly assessing and discussing one’s line of argument in light of quality criteria such as the above

The criteria have been introduced and their importance illustrated in the review of the exemplary article; space precludes further elaboration here. Our intention is neither to enter the discussion on prostitution, nor to debate a specific article or author. The article and its topic were selected solely as a starting point for a discussion of goodness in bioethics, by identifying and presenting potential quality criteria for normative bioethics. One may of course object that the Editor-in-Chief has made a bad judgement in his appraisal of the selected article, or that we should have selected a series of excellent and canonical articles for the analysis. As indicated in the introduction, there are good reasons to believe that the Editor-in-Chief is well qualified. Moreover, finding a set of “excellent and canonical” articles may generate a debate in itself – partly presupposing what we want to obtain, i.e., quality criteria. Nonetheless, we believe to have provided an example of a methodology that may be pursued for other articles as well, and very much welcome such endeavors.

Although we agree with many of the criteria for good bioethics identified in the literature, we have tried to add and to pursue more specific criteria. It is of course still debatable how practically applicable such criteria are and for what purpose (see below). There may also be disagreement on how they should be interpreted and applied. We are open to such criticism and we strongly encourage discussions, revisions, and refinements of the criteria. However, formulating quality criteria for assessing quality criteria is beyond the scope of this article.

It could also be maintained that applying the elaborated criteria may make bioethics less challenging and more boring. We certainly agree with this. It could also be argued that provocation and controversy are crucial quality criteria for bioethics. With this we disagree, especially if such features reduce the trust in bioethics. We believe that trustworthiness is more important for constituting and consolidating bioethics as a professional field than controversy and provocation [30], but acknowledge that others may think otherwise.

It is also interesting to notice that several of the quality criteria were identified by their absence in the analyzed article. Again, this may not be because the article used as an example is particularly bad. All articles – even those published as feature articles in renowned journals – have their strengths and weaknesses. We could have chosen any article in ethics – obviously also our own articles. The point here has been not to pick an arbitrary article, but precisely one that is acclaimed for its quality. The analysis has brought forth interesting quality criteria, indicating that our approach of critical reading can be a tenable way of inventing further, and potentially more rigorous and specific criteria if applied to a larger set of articles.

A reason why we have been able to identify quality criteria by their absence more than by their presence, we believe, is because it is in general easier to identify what is wanting and negative than to identify what is present and positive [2]. This seems to hinge on a basic asymmetry in ethics [31]: it is easier to identify what is bad than what is good. Indeed, this asymmetry might also be one of the reasons why there is little agreement on what is “good bioethics”. Accordingly, it can be as fruitful to tease out quality criteria for “good bioethics” from negative examples or from the neglect of such criteria as from exemplary cases. Our continuous striving for improving bioethics as a methodology and a professional field may advance also by scrutiny of “bad bioethics.”

We should also make reservations about our analysis of the exemplary article. If the author’s only intention is to argue that *if* we accept casual sex and *if* we are consequentialists with a specific view on harm and wellbeing, *then* we may not have as good arguments against prostitution as we think, then our interpretation is wrong. But so are others’ [15–17]. This means that we sincerely have to apologize for our misreading. However, it also means that the article has much less sway and does much less heavy lifting than we have thought. Moreover, it does not mean that the quality criteria elaborated are mistaken. Correspondingly, the author may argue that the exemplary article is not within the field of applied philosophical bioethics, but some other discipline where the quality criteria we identify and discuss are completely irrelevant. Again, this may be as it wants (and thereby undermine the raison d’etre of the content of the exemplary article), yet the quality criteria that are identified in our analysis may still have relevance to applied philosophical bioethics and beyond.

### Specific challenges for radical consequentialist bioethics

One may speculate that the quality criteria discussed be particularly relevant for bioethics in the tradition which the analyzed exemplary article belongs to. Arguably, the present article exemplifies a *radical consequentialist* approach to bioethics. This approach is ‘radical’ for typically challenging prevalent moral views and intuitions, and perhaps in particular deeply culturally entrenched mores and views thought to be lingering remnants of religious morality. The selected article fits this pattern. As discussed above, although the author does not declare allegiance to consequentialism there are clear signs of the approach being a consequentialist one (in particular, singling out ‘harm’ and ‘benefit’ as the sole criteria of interest and attempting to weight these; the author’s admission that the argument “is likely to do more work for consequentialists”).

It seems likely that an analysis of an article written in this paradigm is suited to bring out potential pitfalls and weak points to which those who write in the same paradigm might be particularly susceptible, and that they therefore ought to beware. We give two brief examples of such potential pitfalls for the radical consequentialist:

First, the consequentialist weighing of benefits and harms mandates meticulous interaction with the empirical research on the topic. A danger, then, is when analysis, summarizing and reporting of the empirical findings fall short of conventional standards for assessment of such research (criterion #7 above). This might particularly be so given that many who do normative bioethics do not have special training in empirical sciences or systematic review of empirical literature. Relatedly, because empirical evidence on many matters of bioethical interest might be patchy and of poor quality, precise and rigorous weighing of harms and benefits might not be possible. In such cases cherry picking of studies without transparent and open weighing sometimes occurs.

Second, consequentialism requires a specific value theory, and no such theory is immune to criticism. The exemplary article espouses a value monism in that ‘harm’, defined as “that which is detrimental to well-being” is singled out as the only relevant consideration. This leads to the exclusion from consideration of any arguments that cannot be cashed out in terms of ‘harm,’ inviting the charge of the consequentialist’s approach as a “moral reductionism” in not being able to give a hearing to some prevalent and intuitive counter-arguments [32] – as per criterion #9 & 10.

By this we certainly do not suggest that analyses employing the paradigm of radical consequentialism are generally poorer than others; the point is merely that there are certain pitfalls to which authors within this paradigm might be more susceptible. We are likely to find other quality criteria for good bioethics when investigating articles in other ethics traditions such as deontology or virtue ethics, and corresponding dangers of which these authors must be aware. Moreover, it should be valuable to explore whether the criteria identified by absence in a consequentialist setting would apply to, say, feminist bioethics. Indeed, we would welcome such studies on articles from other paradigms and applied to different fields. We have tried to indicate a way to highlight and elaborate quality criteria in bioethics and encourage further exploration and discussion.

As pointed out in the introduction, the question of “good for what,” underlines that quality criteria may depend on form, content, discipline, context, and purpose. Some criteria may be good for some purposes in some contexts within specific (sub)disciplines. Although we have limited our analysis to a specific interpretation of applied philosophical bioethics (including its empirical underpinnings), we hope that the results can be of some relevance to other fields and disciplines as well. Hence, not only whether the identified criteria are relevant, but also why, how, and where they apply belongs to a further stimulating debate to which we have tried to make a modest contribution.

## Conclusion

Through our analysis of an article that is acclaimed for being exemplary for “good bioethics” we have identified a series of potential quality criteria for bioethics in general and for radical consequentialist bioethics in particular. We have limited our analysis to applied philosophical bioethics and do not claim that the identified quality criteria are universal or absolute, but rather that they may be input to a continuous debate on what is “good bioethics,” both within this specific discipline of bioethics and hopefully beyond. After such a debate, some of the identified criteria may turn out to be of more profound value than others and can be part of defining and improving the field of bioethics. Moreover, we hope that the approach that we have applied can inspire others to identify other quality criteria, and that it can spur a general debate on the quality of bioethics as well as whether we can define context specific and more general quality criteria for the field, as well as whether specific criteria are particularly pertinent for specific subfields of bioethics.

## Additional file

Additional file 1: **Figure S1.** Results from literature search. (DOC 59 kb)

## Footnotes

1 Details of literature search (search terms and search results) are given in Additional file 1.

2 Additionally, one would need to debate whether such general criteria are obtainable or even useful at all. Some criteria may well apply only to certain types of bioethics, such as empirical, anthropological, historical or sociological bioethics, while others may be of a more general kind. Our point is that before starting on such analyses and debates, we need to have a wide range of criteria on the table.

3 «Prostitution» and «prostitute» are controversial terms and it is often argued that «sex work» and «sex worker» are to be preferred. In this paper we have chosen to apply the terms used in the original acclaimed article.

4 The selected article was acclaimed for being an example of good “medical ethics.” Although we do acknowledge that there is an interesting and relevant debate on the relationship between bioethics and medical ethics, entering this debate is beyond the scope of this article.

5 In Moen’s own words: “engaging casually in an activity that has the potential for romantic significance needs not destroy that activity’s romantic significance on other occasions.” No evidence for this statement is given. The significant difficulties in establishing steadfast romantic relationships for prostitutes is one of the points in the research of Edlund and Korn [29], which Moen refers to for other purposes.
