# Supplementary material for: In pursuit of goodness in bioethics: analysis of an exemplary article
Source: BMC Med Ethics. 2018 Jun 15;19:60. doi: 10.1186/s12910-018-0299-9 (PMC6003140; doi:10.1186/s12910-018-0299-9)
Supplement: Supplementary file 1 — Figure S1. Results from literature search. (DOC 59 kb) [file 12910_2018_299_MOESM1_ESM.doc]

**Literature search for “good medical ethics” August 2017:**

**Database: Ovid MEDLINE(R) Epub Ahead of Print, In-Process & Other Non-Indexed Citations, Ovid MEDLINE(R) Daily and Ovid MEDLINE(R)**

**Time: <1946 to Present> (August 2017)**

**Search Strategy**:

--------------------------------------------------------------------------------

1 (good medical ethic* or good ethic* or good clinical ethic* or good bioethic* or good normative ethic* or (goodness adj2 (ethic* or bioethic*))).tw,kf. (134)

2 ((medical ethic* or bioethic* or normative ethic* or clinical ethic*) adj3 (criteria or quality or analysis or analyzis or theor* or normative or framework* or philosoph*)).ti,kf. (248)

3 (*bioethical issues/ or *ethical analysis/ or *ethical theory/ or *ethics, medical/) and normative.tw,kf. (276)

4 (normative and theor* and (ethic* or bioethic*)).tw,kf. (240)

5 ((quality adj3 (ethic* or bioethic*)) or (criteria adj3 (ethic* or bioethic*))).ti. (226)

6 or/1-5 (1003)

7 limit 6 to (english language and yr="2007 -Current" and journal article) (442)

**Scopus**[**https://www.scopus.com/**](https://www.scopus.com/)

**TITLE**(("medical ethic*" OR bioethic* OR "normative ethic*" OR "clinical ethic*") AND (quality OR good* OR theor* OR analyz* OR criteria)) limited to 2007 – 2017, English language (154)
**TITLE-ABS-KEY**(("good medical ethic*" OR "good bioethic*" OR "good normative ethic*" OR "good clinical ethic*") ) avgrenset til 2007-dato, English language (42)

**523 identified references after excluding duplets**

**390 references excluded**

based on title

**133 abstracts assessed**

**102 excluded**

on basis of abstract content

**31 references assessed on content**

**5 references excluded**

on basis of content

**26 studies included of which:**

2 is explicit about criteria

24 mention or discuss criteria implicitly

**Figure S1** Results from literature search
